# Assessment of balance control mechanisms in patient populations using perturbation-based modeling: a narrative review with clinical implications

**DOI:** 10.3389/fnhum.2026.1768417

**Published:** 2026-05-28

**Authors:** Jennifer L. Brodsky, Jae W. Lee, Laurie A. King, Robert J. Peterka

**Affiliations:** 1 National Center for Rehabilitative Auditory Research (NCRAR), Veterans Affairs Portland Health Care System, Portland, OR, United States; 2 Department of Neurology, Oregon Health and Science, Portland, OR, United States

**Keywords:** balance control, biomechanics, clinical populations, closed loop system, motor activation, sensorimotor impairment, sensory reweighting, system identification

## Abstract

Clinical assessments of balance often rely on indirect measures that offer limited insights into the underlying sensorimotor control mechanisms. Consequently, there is a need for standardized and objective quantification of balance control mechanisms. Research over the past 20 years has demonstrated that Central Sensorimotor Integration (CSMI)-type tests can reliably identify functionally relevant parameters accounting for experimentally observed dynamics of the balance control system. However, the complexity and the lack of standardization of this approach have led to heterogeneous methodologies and results, limiting the clinical adoption of CSMI-type testing. Thus, the objective of this narrative review is to explore the application of CSMI-type tasks in quantifying balance control across various clinical populations. We identified articles in PubMed and IEEE Xplore that utilized CSMI or similar methods, specifically those utilizing continuous, wide-bandwidth, pseudorandom surface or visual stimuli and those using linear feedback models of ankle joint control. A total of 18 articles were identified, covering older adults, and individuals with attention deficit hyperactivity disorder, cataracts, heredity spastic paraplegia, lumbar spinal stenosis, mild traumatic brain injury, Parkinson's disease, peripheral neuropathies, and vestibular losses. We outline considerations for interpreting results across studies and recommend steps for standardizing CSMI-type testing to facilitate its implementation in clinical settings, aiming to better understand mechanistic deficits in balance control and to target rehabilitation.

## 1 Introduction

Falls and fall-related injuries are prevalent among older adults and individuals with various diseases. Changes in visual, vestibular, and somatosensory systems and/or neuromuscular control are likely contributors to falls across populations (Horak et al., 1989; Horak, 2006). However, the specific factors contributing to impaired balance control can be difficult to disentangle, particularly if the changes are due to central nervous system dysfunctions, such as in people with Parkinson's disease or traumatic brain injury (TBI). Clinical assessments of balance control often rely on indirect measures of balance, such as spontaneous sway, which offer limited insights into the underlying mechanisms of balance control. An improved understanding of the sensory and neuromotor interactions for balance control, and how they are altered by age and disease, can guide targeted treatment strategies.

### 1.1 Conventional balance assessment methods

Early assessments of balance control relied on qualitative observations of spontaneous sway, such as the Romberg test, which identifies sensory deficits by comparing spontaneous sway with eyes open and closed (Lanska, 2002). Advances in technology allowed quantitative analysis of spontaneous sway by recording the body's center of pressure via force transducers in a platform. For example, Prieto et al. (1996) described quantitative measures of spontaneous sway in both younger and older adults using a wide variety of time- and frequency-domain measures and found that the mean velocity, total power, and 95% power frequency could differentiate spontaneous sway seen in older adults (Prieto et al., 1996). Additional advanced methods have been developed to analyze spontaneous sway, such as stabilogram diffusion analysis (Collins and De Luca, 1993) and entropy measures (Richman and Moorman, 2000; Cavanaugh et al., 2005).

Another method of investigating balance control is through the manipulation of sensory inputs using Computerized Dynamic Posturography (CDP) systems. One common standardized test is the Sensory Organization Test (SOT), which evaluates an individual's ability to maintain balance under six sensory conditions. These conditions include standing on stable, or sway-referenced platform or visual surrounds, thereby systematically altering proprioceptive and visual inputs, respectively (Nashner et al., 1982). Due to the high cost and immobility of CDP systems, the Clinical Test of Sensory Interaction of Balance was developed (Shumway-Cook and Horak, 1986), which uses compliant foam surface and a visual conflict dome placed on the head, instead of using movable platform and visual surrounds. Recent advances in inertial sensors and virtual reality enhanced quantification of modified Clinical Test of Sensory Interaction of Balance (mCTSIB) and accessibility of the Sensory Organization Test (Freeman et al., 2018; Gera et al., 2018; Grove et al., 2021; Watson and Trudelle-Jackson, 2021).

### 1.2 Stimulus-response balance assessment

However, it has been recognized that quantitative identification of balance control dynamics requires the application of a known external disturbance and then an analysis that relates the evoked balance response to the applied disturbance (van der Kooij et al., 2005; Pasma et al., 2014). Disturbances can target visual (van Asten et al., 1988), proprioceptive (Allum, 1983; van der Kooij et al., 2005; Pasma et al., 2014), and/or vestibular (Nashner and Wolfson, 1974) systems. External disturbances are often delivered as transient impulses to examine an individual's ability to react and stabilize the body. For example, the Motor Control Test, conducted using a CDP system, examines the timing and adaptation of neuromotor responses to transient platform translations or rotations (Nashner, 1977). Although transient perturbations provide insights into reactive balance control, they give limited insight into the continuous control required to maintain balance during stance. Thus, it is also crucial to examine sway responses evoked by a continuous externally applied disturbance such as multi-sine (van Asten et al., 1988) or pseudorandom signals (Ishida and Imai, 1980). Early works involving continuous pseudorandom perturbations demonstrated that when stimulus amplitudes are kept sufficiently low, they remain unnoticed by the participants, hence minimizing their effects on typical balance control during quiet stance (Peterka et al., 2018; see Peterka, 2002 for detailed description of the continuous pseudorandom signal characteristics). The evoked sway response can then be analyzed to quantify the dynamic characteristics of the system using time domain measures, such as sway amplitude or velocity. Alternatively, frequency domain measures, such as frequency response functions (FRFs), can be used to characterize responsive sensitivity (i.e., gain) or timing (i.e., phase) as a function of stimulus frequency. However, it has been demonstrated that the balance control strategy elicited is dependent on stimulus parameters, for example causing altered sensitivity to balance disturbances. Therefore, we documented the stimulus parameters that were used in the studies that we summarize. Additionally, Section 4.1 of the Discussion provides guidelines to facilitate comparisons between studies that employed different stimulus conditions.

### 1.3 Model assisted assessment of balance

**Figure 1:**
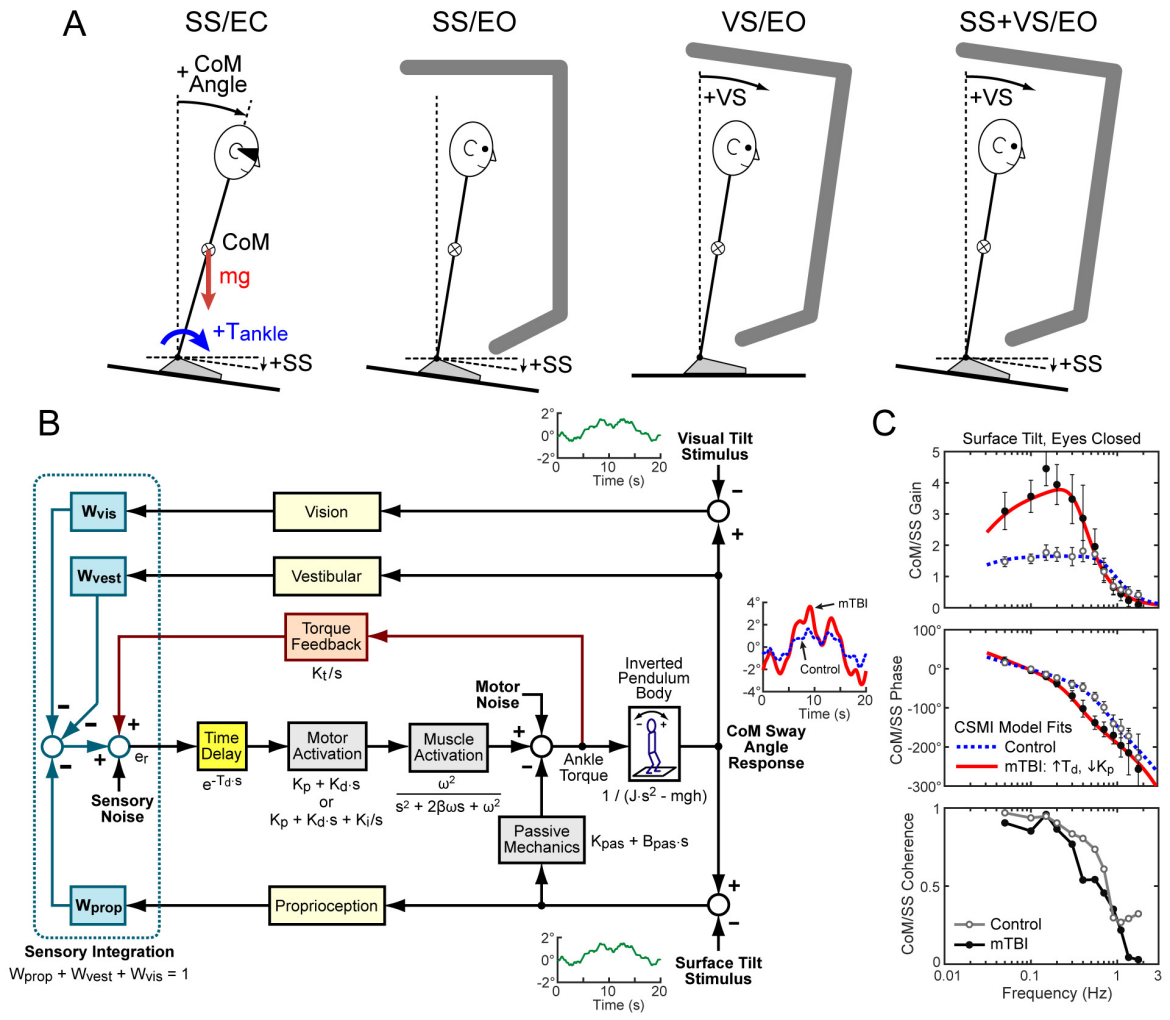
Panel **A** shows stick figure diagrams of four postural control test conditions: support surface/eyes closed (SS/EC), support surface/eyes open (SS/EO), visual surround/eyes open (VS/EO), and combined support surface and visual surround/eyes open (SS+VS/EO), each illustrating center-of-mass (CoM), ankle torque (Tankle), and body orientation (CoM angle). Panel **B** depicts a block diagram of a computational model for sensory integration and motor response in postural control, including pathways for sensory input, sensory noise, motor activation, muscle activation, passive mechanics, ankle torque, and feedback mechanisms, with time-series plots for stimulus and response. Panel **C** presents graphs comparing control and mild traumatic brain injury (mTBI) participants for CoM to surface stimulus gain, phase, and coherence across stimulus frequencies. Modified from Figure 1 in Campbell et al. (2022a) under the Creative Commons Attribution License (CC BY).

The balance control system can be further quantified using a model-based interpretation of the experimentally measured dynamic characteristics, as represented by FRFs. Specifically in this review, we will focus on studies that made use of what we refer to as the Central SensoriMotor Integration (CSMI) model shown in Figure 1B, a version of which was first developed by Peterka (2002). Additionally, this review focuses only on studies that used stimuli that rotate the stance surface and/or the visual scene (Figure 1A) since these stimuli allow for the estimate of the sensory system contributions to balance control. As opposed to surface translations, which introduce redundant sensory information because all sensory systems are simultaneously activated, rotational stimuli allow for targeted manipulation of ankle proprioceptive feedback (via a rotating surface) and visual feedback (via a rotating visual scene). This enables the mathematical separation of the relative contributions of the affected and intact sensory systems. The model output is the rotation angle of the body center of mass (CoM) about the ankle joint. The general form of the model can apply to other stimuli such as surface translations and forces applied to body segments (van der Kooij et al., 2005), but analysis of sway responses to these stimuli do not provide information about sensory contributions because all sensory systems are simultaneously activated.

The CSMI model is a “gray-box” model in that it assumes an internal structure that includes components that are presumed to be functionally meaningful (Olsson et al., 2023). Specifically, the structure includes a “Sensory Integration” mechanism that sums a linear weighted combination of sensory cues from vision, vestibular, and proprioceptive systems. The sensory-derived measure of body orientation is used to generate a corrective ankle torque via a “Motor Activation” mechanism (also referred to as a “Neural Controller” in some studies) that is needed to compensate for destabilization due to gravity and externally applied disturbances. The ankle torque produces CoM body sway with the body modeled as an inverted pendulum. The body motion is sensed by the visual system (sensing body motion relative to the visual scene), the vestibular system (sensing body motion in space), and the proprioceptive system (sensing body motion relative to the stance surface). The overall system is organized as a linear negative feedback-control system with an additional time delay parameter that accounts for signal transmission, central processing, and muscle activation delays. Variations on this basic CSMI model exist that include a “Torque Feedback” mechanism, “Passive Mechanics” that represent muscle/tendon properties that generate corrective torque with no time delay, and/or “Muscle Activation” dynamics that represent the transformation from a muscle efferent signal to ankle torque. The Supplementary material provide additional detailed information about the CSMI model components and parameters, parameter estimation methods, and variations of the basic CSMI model that have been used in different studies.

While the block diagram in Figure 1B provides a conceptual framework for understanding the relationship among components and mechanisms contributing to balance control, the various component blocks also represent explicit mathematical relationships (i.e., transfer functions) that define how the input to each block is transformed by the mathematical properties of that block to produce the output. The input-output relationship can be as simple as a multiplication (e.g., the Sensory Weights) or can represent a differential equation (e.g., the inverted pendulum body dynamics and the Motor Activation mechanism). The mathematical properties of all the blocks can be combined to form a single equation that defines a model-based prediction of the frequency domain FRF. An optimization algorithm is used to adjust model parameters to optimally account for the experimental FRF (example fits shown in Figure 1C). The identified parameters (such as Sensory Weights, Time Delay, and Motor Activation parameters) provide a concise representation of a person's balance control system with the various parameters corresponding to functionally meaningful components of the balance system. This review will focus on the major parameters that account for the dynamic properties of balance control and are commonly reported in studies that use CSMI methods. These parameters are the proprioceptive, visual, and vestibular Sensory Weights (*W*_*prop*_, *W*_*vis*_, and *W*_*vest*_), respectively, Time Delay (*T*_*d*_), and Motor Activation stiffness (*K*_*p*_) and damping (*K*_*d*_). Depending on the CSMI model configuration, the Motor Activation integral control factor (*K*_*i*_) and Torque Feedback parameter (*K*_*t*_), which similarly account for FRF dynamics at low frequencies, will be discussed as well as Passive Mechanics stiffness (*K*_*pas*_) and damping (*B*_*pas*_) parameters.

It may seem surprising that a relatively simple model could account for the experimentally observed dynamics of the balance control system given the true biomechanical complexity (e.g., non-linear multi-segmental body dynamics), neurophysiological complexity (e.g., peripheral sensory system structure/function, spinal and central nervous system processing), and neuromuscular properties (e.g., muscle/tendon mechanics and muscle activation dynamics). Nevertheless, the CSMI model has been shown to account quite well for balance control dynamics which likely arose from the evolution-driven need to satisfy basic physical principles, such as the requirement to maintain the body CoM over the base of support, and the utility of responding proportionally to balance disturbances with minimal energy expenditure.

The purpose of this narrative review is to explore the application of CSMI-type methods (i.e., using wide bandwidth sensory perturbations combined with model-based interpretations) to quantify balance control properties across various clinical populations. We begin by outlining the methodology of the literature search to find studies that used CSMI-type methods. We then focus specifically on the results of research using CSMI and similar methods across different clinical populations. To the limited extent possible, we also compare results from CSMI methods with those from conventional balance assessments applied to similar clinical populations. The paper concludes by discussing the potential for application of this technique in a clinical setting and limitations of this method including interpretation of results across studies with differing protocols.

## 2 Methods

Article identification began with a review of articles known to the authors R.J.P., J.W.L., and J.L.B. that utilize CSMI or similar methods to quantify balance control. A PubMed search was performed using search terms previously used in an article by Olsson et al. (2023) that focused on models used to characterize standing balance control in older adults. The PubMed search was: “(((“postural balance”[MeSH Terms] OR “posture”[MeSH Terms]) AND “humans”[MeSH Terms]) AND “models,” [MeSH Terms]) AND control[Title/Abstract].” Additionally, an IEEE Xplore search was conducted using the search terms “((“Full Text & Metadata”:balance^*^ AND “Full Text & Metadata”:human^*^ AND “IEEE Terms”:biomechanic^*^ AND “IEEE Terms”:control^*^)).” The resulting articles were then screened by J.L.B. and J.W.L. for inclusion first by title, then abstract, and finally the full article. Articles were excluded if they did not use human subjects, lacked a closed-loop “gray-box” model of balance control, lacked the use of wide-bandwidth sensory perturbations to quantify balance control, did not use rotational stimuli of the stance surface or visual surround, or did not report model-based parameters such as Sensory Weights, Time Delay (*T*_*d*_), or Motor Activation (e.g., *K*_*p*_ and *K*_*d*_). Articles were also excluded if they only assessed balance control in healthy younger subjects. Articles from the authors' personal libraries, additional articles identified by PubMed and IEEE searches, and articles identified by searching the last authors' bibliographies were included if they met the review criteria. Risk of bias was assessed using the adapted Newcastle-Ottawa Scale for risk of bias, adapted for cross-sectional studies (Carra et al., 2025), by J.L.B and J.W.L with disagreement settled through discussion.

## 3 Results

Article identification, screening, and review resulted in a final total of 18 articles that met the inclusion criteria. These studies are listed by diagnosis in Table 1, along with the sensory test conditions used and the components of the CSMI model included in each study. In addition to older adults, other populations tested using CSMI-type methods include attention deficit hyperactivity disorder (ADHD), cataracts, hereditary spastic paraplegia (HSP), lumbar spinal stenosis, mild traumatic brain injury (mTBI), Parkinson's disease, various peripheral neuropathies, and different levels of vestibular loss.

**Table 1:** Publications using CSMI methods with population descriptions, test conditions, and peak-to-peak stimulus amplitudes, model components.

References	Population description	N	Test conditions					Model components				RoB
			SS	SS	VS	SS+VS	Stimulus amp (peak-to-peak)	Motor act	Passive mech	Torque FB	Muscle act	NOS-xs score
			EC	EO	EO	EO						
Peterka (2002)	Bilateral vestibular (severe)	4	X	X	X	X	1°, 2°, 4°	PID	X			4
	Healthy controls	8	X	X	X		0.5°,1°, 2°, 4°, 8°					
Cenciarini et al. (2010)	Older adult	7	X				4°	PID	X			4
	Young adult	10										
Peterka et al. (2011)	Unilateral vestibular (complete)	11	X				1°, 2°, 4°, 8°	PD	X	X		4
	Healthy controls	11										
Pasma et al. (2015)	Older adult	10		X			0.5°, 1°	PD		X	X	5
	Young adult	10										
	Cataracts	10										
	Polyneuropathy	10										
Wiesmeier et al. (2015)	Older adult	20	X	X			0.5°, 1°	PID	X			4
	Middle age adult	16										
	Young adult	19										
Wiesmeier et al. (2017)	Older adult	35	X	X			0.5°, 1°	PID	X			2
	Middle age adult	35										
van Kordelaar et al. (2018)	Unilateral vestibular (acute, partial)	11	X				0.5°, 1°	PD		X		4
	Healthy controls	12										
Feller et al. (2019)	Parkinson disease	8	X	X	X		1°, 2°, 4°	PD		X		4
	Healthy controls	8										
Jansen et al. (2019)	Attention deficit hyperactivity disorder	10	X	X			0.5°, 1°	PID	X			6
	Healthy controls	10										
Kneis et al. (2019)	Lumbar spinal stenosis	11	X	X			0.5°, 1°	PID	X			3
	Healthy controls	15										
Kneis et al. (2020)	Chemo-induced peripheral neuropathy	8	X	X			0.5°, 1°	PID	X			6
	Healthy controls	15										
Waibel et al. (2021)	Chemo-induced peripheral neuropathy	31	X	X			0.5°, 1°	PID	X			6
	Healthy controls	36										
Campbell et al. (2022a)	mTBI/concussion	52	X	X	X	X	2°, 4°	PD		X		6
	Healthy controls	58										
Campbell et al. (2022b)	mTBI/concussion	31	X	X	X	X	2°	PD		X		5
Dalin et al. (2023)	Hereditary spastic paraplegia	9	X	X			0.5°, 1°	PID	X			3
	Healthy controls	9										
Corre et al. (2024)	Unilateral vestibular (partial)	15	X	X	X		2°	PD		X		4
	Bilateral vestibular (severe)	17										
Healthy controls	20										
Campbell et al. (2025)	mTBI/concussion	153	X	X	X	X	2°	PD		X		8
Neumann et al. (2025)	Older adult Younger adult	40 40			X^*^			PD		X		5

EC, eyes closed condition; EO, eyes open; NOS-xs, Newcastle-Ottawa risk of bias Scale adaptation for cross sectional studies (0–3, high; 4–6, moderate; 7–9, low); RoB, risk of bias; SS, support surface stimulus; VS, visual surround stimulus.

^*^Foam surface used to reduce proprioceptive contribution.

Motor Activation: PD, proportional-derivative control; PID, proportional-integral-derivative control.

All the studies identified a basic set of model parameters (i.e., Sensory Weights, *T*_*d*_, *K*_*p*_, and *K*_*d*_). Additional model components were included in many studies (Table 1 and Supplementary material). Many studies used a limited set of sensory test conditions with only six out of 18 using visual perturbations that allowed measurement of the visual sensory weight (Peterka, 2002; Feller et al., 2019; Corre et al., 2024; Campbell et al., 2025, 2022a,b). Stimulus amplitudes were not consistent across studies with peak-to-peak amplitudes ranging from 0.5° to 8°. Most studies used stimuli that evoked anterior-posterior (sagittal plane) sway but two used stimuli that evoked mediolateral (frontal plane) sway (Cenciarini et al., 2010; Peterka et al., 2011).

Five studies were identified that compared older adults to middle-aged or younger adults (Cenciarini et al., 2010; Pasma et al., 2015; Wiesmeier et al., 2015, 2017; Neumann et al., 2025). Of these, one study compared older adults to younger adults (Pasma et al., 2015), one made comparisons between older, middle-aged, and younger adults (Wiesmeier et al., 2015), and one compared older to middle-aged adults and also examined the effects of balance training (Wiesmeier et al., 2017). Neumann et al. (2025) used only a visual stimulus generated through virtual reality goggles while participants stood on foam to compare older to younger adults, and also assessed the test-retest reliability of their CSMI protocol. The fifth study utilized mediolateral perturbations that compared older to younger adults (Cenciarini et al., 2010). The pooled number of older adults from these studies was 112, and the pooled number of middle age or younger adult controls was 130.

People with varying severity and chronicity of vestibular loss were tested in four studies (Peterka, 2002; Peterka et al., 2011; van Kordelaar et al., 2018; Corre et al., 2024). Corre et al. (2024) performed testing on patients with chronic, partial unilateral vestibular loss (UVL; *n* = 15) and severe bilateral vestibular loss (BVL; *n* = 17) compared to 20 healthy controls. van Kordelaar et al. (2018) examined 11 people with acute, partial UVL compared to 12 controls. Peterka (2002) compared four people with chronic, severe BVL to eight healthy age-matched controls. The effects of complete, chronic UVL on CSMI performance were examined by Peterka et al. (2011) in 11 people with UVL compared to 11 controls using pseudorandom perturbations that evoked mediolateral sway.

Mild TBI (mTBI) was a diagnosis in three studies. Campbell et al. (2022a) examined people with mTBI with chronic symptoms compared to a healthy control group. There were two other studies by the same research group who used CSMI testing that evaluated the effectiveness of rehabilitation procedures. In one study, the effectiveness of traditional rehabilitation vs. rehabilitation enhanced using audio biofeedback was evaluated (Campbell et al., 2022b). A second study used CSMI testing in people with mTBI in the subacute stage, 2 weeks to 6 months post-injury in a clinical trial that evaluated early vs. delayed physical therapy (Campbell et al., 2025). The pooled number of people with mTBI, was 236, and healthy controls was 58.

Two studies used the same testing protocol to examine people with chemotherapy-induced peripheral neuropathy (CIPN) compared to matched controls (Kneis et al., 2020; Waibel et al., 2021). These studies provide comparisons for a total of 39 people with CIPN and 51 healthy age, gender, height, and weight matched controls. Both studies also used CSMI results to evaluate changes following exercise therapies.

There are single studies investigating CSMI responses for ADHD (ADHD = 10, controls = 10; Jansen et al., 2019), older adults with cataracts (cataracts = 10, older adult controls = 10; Pasma et al., 2015), hereditary spastic paraplegia (HSP = 9, controls = 9; Dalin et al., 2023), lumbar spinal stenosis (stenosis = 11, controls = 15; Kneis et al., 2019), Parkinson's disease (Parkinson's = 8, controls = 8; Feller et al., 2019), and older adults with polyneuropathy (polyneuropathy = 10, older adult controls = 10; Pasma et al., 2015).

There are no published normative values for the CSMI test. Due to this, the differences in the test protocols, and the differences in components included in CSMI models, nearly all studies (16 of 18) included healthy controls for comparison with their patient group. Table 2 summarizes only the studies that directly compared differences in CSMI parameters between control and patient groups. The 2 studies not included in Table 2 (Campbell et al., 2025, 2022b) did not directly have control groups and instead used previously published CSMI data to contextualize their intervention findings.

**Table 2:** Differences in various patient populations compared to healthy controls for CSMI model parameters.

Population description	Study count	Proprioceptive weight (*W_*prop*_*) eyes closed	Proprioceptive weight (*W_*prop*_*) eyes open	Visual weight (*W_*vis*_*)	Time delay (*T_*d*_*)	Stiffness (*K_*p*_*)	Damping (*K_*d*_*)	Integral gain (*K_*i*_*) or torque FB (*K_*t*_*)
Aging	5	↑^**a**^ NR = = NR	NR ↑↑↑NR	NR NR NR NR↑	=^**a**^ ↑↑↑=	↑^**a**^ ↓↓↓=	↑^**a**^ ↑= = =	↑^a^ = ↓↓=
UVL	3	= ↑^**a**^ ↑^b^	= NR NR	= NR NR	= =^**a**^ =^b^	= =^**a**^ ↑^b^	= =^**a**^ =^b^	NR = ↓
BVL	2	↑↑	↑↑	↑↑	↑^c^ ?^d^	↑^e^ ?^f^	= =	NR ?
CIPN	2	↓=	NR NR	NR NR	= =	= =	= ↓	= ↑
mTBI	1	=	=	↑	↑	↓^g^	↓^g^	↑^e^
ADHD	1	=	=	NR	↑	=	=	↓
Cataracts	1	NR	↑	NR	↑	↓	=	=
HSP	1	=	NR	NR	↑	=	=	↓
LSS	1	↓	NR	NR	=	=	=	=
Parkinson's disease	1	=	NR	NR	=	=	=	=
PN	1	NR	=	NR	↑	↓	=	=

Populations: ADHD, attention-deficit hyperactivity disorder; BVL, bilateral vestibular loss; CIPN, chemotherapy-induced peripheral neuropath; HSP, hereditary spastic paraplegia; LSS, lumbar spinal stenosis; mTBI, mild traumatic brain injury; PN, polyneuropathy.

Results: NR, results not reported; ↑, higher in patients; ↓, lower in patients; =, no difference; ?, mixed results. Symbols represent results in order of listed references.

^*a*^Study that used perturbations that evoked frontal plane sway.

^*b*^Patients classified as having acute vestibular dysfunction with evidence for asymmetric function.

^*c*^Time delay larger on support surface but not visual surround perturbations.

^*d*^Mixed results with 2 of 4 BVL showing greater time delay, and 2 showing shorter time delay compared to controls.

^*e*^Larger on visual surround but not support surface perturbations.

^*f*^Mixed results with 2 of 4 BVL showing higher stiffness and one with lower stiffness compared to controls.

^*g*^Lower stiffness and damping only on support surface perturbations.

Reference orders: aging; Cenciarini et al. (2010); Pasma et al. (2015); Wiesmeier et al. (2015), 2017); Neumann et al. (2025). UVL; Corre et al. (2024); Peterka et al. (2011), van Kordelaar et al. (2018). BVL; Corre et al. (2024); Peterka (2002). mTBI; Campbell et al. (2022a). CIPN; Kneis et al. (2020); Waibel et al. (2021). ADHD; Jansen et al. (2019). Cataracts; Pasma et al. (2015). HSP; Dalin et al. (2023). LSS; Kneis et al. (2019). Parkinson's disease; Feller et al. (2019). PN; Pasma et al. (2015).

### 3.1 Sensory weights (*W_*prop*_, W_*vis*_*)

When comparing older adults to younger or middle-aged adults, older adults had larger *W*_*prop*_ on surface-tilt, eyes-open tests in all four studies where this was investigated (Cenciarini et al., 2010; Pasma et al., 2015; Wiesmeier et al., 2015, 2017). When tested with eyes closed, the two studies by Wiesmeier et al. (2015), which included the same subjects on both eyes open and eye closed tests, found no effect of age on *W*_*prop*_. Using only a visual stimulus, Neumann et al. (2025) found increased *W*_*vis*_ in older adults compared to younger controls. However, all participants were tested while standing on a foam pad, which is commonly used to reduce the ability to use proprioceptive-driven postural control responses and may have influenced the sensory weighting. One other study (Cenciarini et al., 2010) found that *W*_*prop*_ was larger in older adults in the eyes closed condition. However, the Cenciarini study, using stimuli that evoked frontal plane sway and a large stimulus amplitude, was much different than the Wiesmeier studies using stimuli that evoked sagittal plane sway and small stimulus amplitudes, making a comparison between these studies problematic.

Unsurprisingly, people with severe bilateral vestibular loss showed greater reliance on proprioception and vision orientation information compared to healthy controls (Peterka, 2002; Corre et al., 2024). Patients with complete UVL (Peterka et al., 2011) and acute vestibular dysfunction (van Kordelaar et al., 2018) showed increased *W*_*prop*_ on surface-tilt eyes-closed tests relative to controls. But those with chronic partial UVL (Corre et al., 2024) had similar *W*_*prop*_ (eyes open and closed tests) and *W*_*vis*_ to controls consistent with compensatory mechanisms being able to mask postural deficits when the vestibular deficit is incomplete. However, when the vestibular asymmetry was complete or very large, CSMI tests identified sensory weight changes years following UVL (Peterka et al., 2011). This contrasts with SOT results which showed eventual return to a normal assessment of balance function following sudden complete UVL (Black et al., 1989).

People with chronic mTBI, when compared to a healthier control group, showed a ~40% larger *W*_*vis*_ on visual stimulus/eyes open tests (mTBI *W*_*vis*_ = 0.151, control *W*_*vis*_ = 0.108), but no differences in *W*_*prop*_ (eyes open and closed) for surface-tilt tests (Campbell et al., 2022a). This increased visual sensitivity is qualitatively consistent with results demonstrating increased sway in mTBI subjects viewing a rotating visual scene while standing on foam (Wright et al., 2022) and with results from an instrumented mCTSIB test where eye closure was associated with a greater increase in sway area in people with mTBI compared to controls (Martini et al., 2023). In contrast, many mTBI subjects using the SOT (Campbell et al., 2021—which included the same subjects reported in the Campbell et al., 2022a) showed multiple abnormal SOT “sensory ratios” suggesting widespread sensory utilization difficulties. However, we note that other CSMI-identified balance control parameters (time delay and motor activation stiffness discussed below) in mTBI subjects can potentially account for the unusually large sways associated with the observed SOT abnormalities.

In a clinical trial with subacute mTBI groups (Campbell et al., 2025), *W*_*vis*_ at baseline was higher (*W*_*vis*_ = 0.133) than control values (*W*_*vis*_ = 0.108) yet lower than chronic mTBI values (*W*_*vis*_ = 0.151) from the 2022a paper. While these findings are a comparison between different studies, they demonstrate that the CSMI may be able to detect differences in sensory weighting between people with mTBI and compared to controls. Unlike testing using the SOT, which often shows abnormalities in multiple measure of sensory utilization (Campbell et al., 2021), the CSMI was able to isolate a visual weighting deficit which could better guide interventions for people with subacute or chronic imbalance after mTBI.

In preliminary work in people with CIPN, Kneis et al. (2020) found that *W*_*prop*_ (eyes-closed surface-tilt tests) was smaller in people with CIPN compared to controls. However, in a later study for an exercise intervention that had a larger sample, Waibel et al. (2021) did not find any difference in *W*_*prop*_ between CIPN and healthy controls at baseline. They point out in their limitations that the disease severity in their CIPN group was less than that of the pilot study by Kneis which may explain why the larger study found contradictory results. This may indicate that, in people with CIPN, there is a threshold level of somatosensory loss that must be reached before CSMI testing is able to detect changes in proprioception utilization. While there are no studies that directly compared CSMI testing to other methods of posturography, a review paper that included the effect of chemotherapy on balance found that multiple studies showed an increased visual reliance for balance in people with CIPN when using posturography such as the SOT or instrumented mCTSIB (Wechsler and Wood, 2021).

In people with Parkinson's disease, Feller et al. (2019) overall found similar *W*_*prop*_ (eyes-closed surface-tilt and visual surround tilt tests) to healthy controls and no differences between results when on and off levodopa medication. But there were some outliers in their limited data set. One participant was an outlier with higher *W*_*prop*_ on and off medication conditions, and another had higher *W*_*prop*_ only when on medication. When comparing *W*_*prop*_ at different stimulus amplitudes, people with Parkinson's disease showed a smaller decrease in *W*_*prop*_ than healthy controls with increasing amplitudes. The outlier results could be due to chance, or to differences in disease progression between the participants that have been shown in SOT testing (Harro et al., 2018). On the SOT, people with less severe PD do not appear different than healthy controls, however at later disease stages, they show lower vestibular and visual ratios (Harro et al., 2018). However, the sensitivity of the CSMI to sensory weighting differences in people with PD remains unclear with only a single study and small sample size. Further studies with a larger sample size and including a range of disease severity are needed to clarify if sensory weighting in people with Parkinson's disease differs from healthy controls or in response to dopaminergic medications.

When comparing *W*_*prop*_ (eyes-open surface-tilt tests) in people with cataracts, Pasma et al. (2015) found that people with cataracts have higher *W*_*prop*_ than both healthy older and young adults. These findings reinforce that reliance on proprioception increases when vision is impaired (Black et al., 2008; O'Connell et al., 2017; Zarei et al., 2023), and that this increase is beyond known age-related increases in proprioceptive reliance for balance (Peterka and Black, 1990).

Kneis et al. (2019) found that people with lumbar spinal stenosis had lower *W*_*prop*_ (eyes-closed surface-tilt tests) compared to controls. This is consistent with disrupted proprioception that has been identified in people with chronic low back pain (Meier et al., 2019).

For the remaining diagnosis groups—ADHD, hereditary spastic paraplegia, and polyneuropathy—no differences were seen on sensory weights compared with controls (Pasma et al., 2015; Jansen et al., 2019; Waibel et al., 2021; Dalin et al., 2023). This is somewhat surprising for polyneuropathy, as the authors note, because prior literature has shown that people with polyneuropathy have decreased use of proprioception for standing balance (Henry and Baudry, 2019).

### 3.2 Time delay (**T**_**d**_)

In older adults, *T*_*d*_ was generally longer compared to young and middle-aged adults for surface-tilt stimuli that evoked anterior-posterior sway (Pasma et al., 2015; Wiesmeier et al., 2015, 2017). However, no *T*_*d*_ difference was seen between older and younger adults when only visual perturbations were used (Neumann et al., 2025), and in one paper that assessed frontal plane balance using eyes-closed surface-tilt tests (Cenciarini et al., 2010). CSMI analysis lumps all sources of *T*_*d*_ into a single parameter. Aging likely impacts multiple aspects of sensorimotor control including changes due to sarcopenia affecting the ability to recruit motor units (Pereira Da Silva Alves et al., 2023) as well as central and peripheral nervous system degeneration (Borzuola et al., 2020).

In subjects with severe bilateral vestibular loss, *T*_*d*_ was significantly increased relative to controls in CSMI tests using surface-tilt perturbations (eyes open and eyes closed; Corre et al., 2024) but an earlier study that included only four patients showed mixed results (Peterka, 2002). There was no evidence for *T*_*d*_ differences between controls and patients with chronic complete (Peterka et al., 2011) or partial UVL (Corre et al., 2024), or acute unilateral loss (van Kordelaar et al., 2018).

For people with mTBI, *T*_*d*_ was larger than in healthy controls (Campbell et al., 2025, 2022a,b). People with chronic mTBI showed significantly larger *T*_*d*_ across all CSMI test conditions compared to controls (Campbell et al., 2022a,b) and were similarly large in people with subacute mTBI (Campbell et al., 2025). It is possible that *T*_*d*_ in mTBI can be attributed to decreased central conductivity from the diffuse axonal injury process.

Other groups that were found to have larger *T*_*d*_ than healthy matched controls are ADHD (Jansen et al., 2019), hereditary spastic paraplegia (Dalin et al., 2023), and cataracts and polyneuropathy (Pasma et al., 2015). In ADHD, the authors attribute the increased *T*_*d*_ to central abnormalities related to hyperactivity/impulsivity. For people with hereditary spastic paraplegia and peripheral neuropathy, increased *T*_*d*_ could be due to impaired nerve conduction of peripheral motor or sensory signals, respectively. Studies examining people with cataracts, CIPN, Parkinson's disease, and lumbar spinal stenosis found no difference in *T*_*d*_ when compared to their respective control groups (Pasma et al., 2015; Feller et al., 2019; Kneis et al., 2019; Waibel et al., 2021).

Regardless of the cause, increased *T*_*d*_ may contribute to loss of balance and/or falls. In a closed-loop feedback control system, increased *T*_*d*_ that is not otherwise compensated can cause poorly controlled oscillatory responses to perturbations and instability (Campbell et al., 2022a). Delays in postural responses during transient perturbations or during walking have been linked to increased risk for falls in older adults (Pijnappels et al., 2010; Batcir et al., 2020). This makes *T*_*d*_ a potential target for rehabilitation for falls prevention.

### 3.3 Motor activation (*K_*p*_, K_*d*_, K_*i*_*), passive mechanics (*K_*pas*_, B_*pas*_*), and torque feedback (*K_*t*_*)

Older adults generally have been shown to have decreased *K*_*p*_ compared to both young and middle-aged adults while results are mixed for *K*_*d*_ (Wiesmeier et al., 2015, 2017; Neumann et al., 2025). These differences are likely to be due primarily, but not entirely, to changes in central and peripheral motor signaling (Borzuola et al., 2020) rather than passive mechanical properties contributing to joint stiffness (Chesworth and Vandervoort, 1989; Aronson et al., 2014). Specifically, in two studies that used a CSMI model that included passive mechanical stiffness (*K*_*pas*_) and damping factors (*B*_*pas*_), these factors were about 1/10th as large as the Motor Activation factors *K*_*p*_ and *K*_*d*_ (Wiesmeier et al., 2015, 2017). However, in these two studies *K*_*pas*_ and *B*_*pas*_ did also decrease with age. In contrast to these results, the study by (Cenciarini et al., 2010) found an increase in *K*_*p*_and *K*_*d*_ with age but no change in *K*_*pas*_ or *B*_*pas*_.

People with chronic mTBI compared to controls had reduced *K*_*p*_ and *K*_*d*_ in all CSMI test conditions that included support surface perturbations, but not with visual perturbations alone (Campbell et al., 2022a,b). In a clinical trial study on people with subacute mTBI (Campbell et al., 2025) their baseline *K*_*p*_ measures were similar to those seen in Campbell et al. (2022a).

Vestibular loss of any degree did not affect *K*_*d*_ (Peterka, 2002; Peterka et al., 2011; Corre et al., 2024). *K*_*p*_ was largely unaffected by vestibular dysfunction with the exception of increased *K*_*p*_ being observed in severe bilateral vestibular loss subjects but only on CSMI visual-tilt tests (Corre et al., 2024) and in subjects with acute vestibular dysfunction (van Kordelaar et al., 2018).

In people with polyneuropathy, Pasma et al. (2015) found they had decreased *K*_*p*_ (but not *K*_*d*_) compared to controls. Similar to mTBI subjects (see below), the *K*_*p*_ decrease may be a compensation for their increased *T*_*d*_.

Among the remaining diagnosis groups, no studies found differences in *K*_*p*_ and *K*_*d*_ compared to controls (Pasma et al., 2015; Feller et al., 2019; Jansen et al., 2019; Kneis et al., 2019; Waibel et al., 2021; Dalin et al., 2023) with the exception of a decreased *K*_*p*_ in patients with cataracts (Pasma et al., 2015) where their *K*_*p*_ was decreased relative to healthy elderly whose *K*_*p*_ was also decreased relative to young subjects.

Decreased *K*_*p*_ accompanied by increased *T*_*d*_ can generally be seen as a feature of aging, mTBI, and polyneuropathy. As discussed by Campbell et al. (2025), 2022a,b) in relation to their mTBI studies, a reduction in stiffness can be understood as providing compensation for the presence of increased *T*_*d*_ since it improves inherent stability but at the cost of having “sloppier” control that results in larger than normal sway in response to a given perturbation. But because body CoM during stance must remain within the base of support, a balance system with long *T*_*d*_ and reduced stiffness, while theoretically inherently stable, is susceptible to instability caused by the CoM being displaced beyond the base of support by modest-sized perturbations thus causing a fall or evoking the need to take a step to maintain balance.

An Integral Control factor (*K*_*i*_) was included in the CSMI model in nine of the 18 studies while the other eight studies included a Torque Feedback gain parameter (*K*_*t*_). A larger value of *K*_*i*_ or *K*_*t*_ accounts for a greater decrease in FRF gain and a larger phase advance with decreasing frequency. A lower FRF gain at low frequencies indicates that the body sway was less affected by the stimulus and therefore remained closer to an upright orientation.

### 3.4 Effects of intervention

Eight of 18 studies used CSMI methods to evaluate changes in balance control arising from various treatments applied to patient populations. Summarized effects of treatments on each model parameter can be seen in Table 3. These included Wiesmeier et al. (2017) who used a balance training intervention on older adults. They found that balance training reduced *W*_*prop*_ in older adults (i.e., a change toward values found in younger adults) while it had no effect on other CSMI parameters. Feller et al. (2019) compared CSMI results in patients with Parkinson's disease on and off medication, with overall no effect, except for two outliers for *W*_*prop*_ as discussed above. Kneis et al. (2019) evaluated the effect of a surgical intervention for lumbar spinal stenosis and found no differences in CSMI model parameters following surgery. Dalin et al. (2023) assessed the effect of treadmill training in people with HSP and found no overall effect on CSMI parameters.

**Table 3:** Effects of interventions on CSMI model parameters in various patient populations.

References	Population description	Intervention	Outcome
Campbell et al. (2022b)	mTBI	Traditional vestibular and balance training with or without auditory biofeedback	Both groups improved on CSMI conditions with support surface perturbations. Time Delay, stiffness, and damping approached normal values with larger effect sizes for the biofeedback group
Campbell et al. (2025)	mTBI	Early or later traditional vestibular and balance training	Early group improved Time Delay and stiffness, while delayed group had worsened Time Delay and stiffness that did not improve following intervention
Dalin et al. (2023)	HSP	Treadmill training	No effect on overall CSMI results
Feller et al. (2019)	Parkinson's disease	Dopaminergic medication	No effect on overall CSMI results, but with two out of eight participants as outliers
Kneis et al. (2019)	LSS	Surgery	No effect on overall CSMI results
Kneis et al. (2020)	CIPN	Balance training	Improved proprioceptive weight
Waibel et al. (2021)	CIPN	Endurance and balance training or endurance training alone	Both groups improved Time Delay and stiffness but not proprioceptive weight
Wiesmeier et al. (2017)	Aging	Balance training	Reduced proprioceptive weight to be more like younger adults

Populations: CIPN, chemotherapy-induced peripheral neuropath; HSP, hereditary spastic paraplegia; LSS, lumbar spinal stenosis; mTBI, mild traumatic brain injury; SS, support surface perturbations.

Interventions for improved balance in CIPN were examined in two studies. Kneis et al. (2020) applied a balance-based exercise therapy in patients with CIPN and found that *W*_*prop*_ improved to be closer to the values of healthy controls. Waibel et al. (2021) compared two interventions (endurance plus balance training vs. endurance training alone) in patients with CIPN. All participants, regardless of interventions, demonstrated improved *T*_*d*_ and *K*_*p*_ over time. No significant effect was found for the type of therapy group. However, the therapy groups differed by age, which was also correlated with the change over time. As discussed earlier, the participants in this study had less severe CIPN with no difference in *W*_*prop*_ from controls.

Two studies in mTBI patients compared the effects of augmenting balance therapy using feedback from body-worn motion sensors to standard therapy (Campbell et al., 2022b) and compared the effectiveness of early vs. delayed balance therapy (Campbell et al., 2025). In both studies, balance and vestibular training for mTBI, *W*_*vis*_ was shown to change toward lower *W*_*vis*_ values following rehabilitation with greater changes in mTBI subjects who received early vs. delayed rehabilitation (Campbell et al., 2025, 2022b). In their study comparing traditional balance and vestibular training to traditional training augmented by audio biofeedback, Campbell et al. (2022b), found improvements in *T*_*d*_ for both groups only in conditions with support surface perturbations. When comparing parameter changes based on timing of intervention, Campbell et al. (2025) found that early physical therapy improved (i.e., reduced) *T*_*d*_. However, in the group where therapy began later, *T*_*d*_ showed little or no improvement. Both traditional and audio-biofeedback enhanced therapy improved *K*_*p*_ and *K*_*d*_, with larger effect sizes seen for the augmented group (Campbell et al., 2022b). Subacute mTBI subjects who received early therapy showed an increase in *K*_*p*_ (toward normal) on CSMI tests that included surface-tilt stimuli while those who received delayed therapy showed additional decrease in *K*_*p*_ and no recovery toward normal following the delayed therapy.

### 3.5 Reliability

The reliability of CSMI-type methods has been assessed in several ways (Pasma et al., 2017; Peterka et al., 2018; Neumann et al., 2025). Peterka et al. (2018) evaluated the methodological quality of using pseudorandom perturbation stimuli by assessing the number of stimuli cycles necessary to obtain reliable parameter estimates. They found that model parameters achieved stable variance at six cycles for support surface perturbations, but 11 cycles were needed for visual perturbations. As discussed in the Supplementary material, Pasma et al. (2017) conducted a sensitivity analysis of the model parameters by comparing model estimates of parameter results with experimental data. Finally, Neumann et al. (2025) assessed the test-retest reliability of a CSMI protocol with 20 older and 20 younger adults using virtual reality to provide visual perturbations while subjects stood on foam. They found intraclass correlation that were high for *W*_*vis*_, *K*_*d*_, and *K*_*p*_ (0.896 to 0.966), but moderate for *T*_*d*_, and low for a torque feedback component. The high reliability of *W*_*vis*_, *K*_*d*_, and *K*_*p*_ are promising, however, replication in other test protocols including support surface perturbations are still needed.

## 4 Discussion

Our review was motivated by the recognition that CSMI-type methods have been used for over 20 years to study balance control in a variety of patient populations and to assess the efficacy of applied treatments. Results have shown potential benefit of the CSMI that allows us to quantify different mechanisms of balance control across varied clinical populations. This could provide information to guide interventions to address abnormal balance specific to each patient population. We hope that this review may serve to motivate other researchers to validate and apply these methods in their own work and to consider the potential for these methods to become a standard clinical tool.

The chief advantage of CSMI methods is the ability to quantify balance control in terms of functionally relevant parameters during steady-state conditions such that balance deficits can be attributed to particular components of the balance system (examples: utilization of sensory cues, delays in generating corrective responses, reduced magnitude of corrective responses). This approach enables us to quantify the continuous control strategies required to maintain upright stance across varying sensory and environmental conditions. The potential exists for the development of therapies that focus on the identified deficit rather than on general balance control. The CSMI methods can obtain sufficiently reliable estimates of parameters from individual subjects (Peterka et al., 2018) such that they can be used to characterize the efficacy of treatments (Wiesmeier et al., 2017; Feller et al., 2019; Kneis et al., 2019, 2020; Waibel et al., 2021; Campbell et al., 2025, 2022b) and to identify subgroups within a patient population (e.g., mTBI; Campbell et al., 2022a). Specifically, in Campbell et al. (2025) the CSMI was used to track the effectiveness of early compared to delayed physical therapy to address balance dysfunction in people with mTBI.

However, it is evident from the information shown in Table 1 (details in Supplementary material) that a standard test protocol is currently not defined since a variety of test conditions have been applied and CSMI models with different components have been used. Given this variety, below we discuss considerations that can be employed to compare results from different studies that used different CSMI models or stimulus conditions, or to recognize when comparisons are likely to be problematic. Finally, we discuss limitations and recommendations for future use of CSMI methodology.

### 4.1 Considerations in across-study comparisons

Below are considerations that can be made when attempting to compare results across studies that used different model structures or different stimulus conditions.

1) FRF gains are known to decline with increasing stimulus amplitude (Peterka, 2002). In the CSMI model, with all else being equal, the sensory weight parameter accounts for the FRF gain decrease with increasing stimulus amplitude and represents the phenomenon of an amplitude-dependent sensory reweighting. Therefore, a direct comparison of Sensory Weight measures across studies without considering what stimulus amplitude was used can lead to erroneous conclusions. To some extent, results from Peterka (2002) can be used to estimate expected amplitude dependent changes since this study analyzed results from a wide variety of stimulus conditions and stimulus amplitudes.
2) The stimulus amplitude-dependent behavior also restricts the type of stimulus needed for accurate identification of model parameters. If the balance system behaved as a linear system, then a set of individual sinusoidal stimuli could be used to measure gain and phase across a wide range of frequencies. However, the non-linear amplitude-dependent behavior means that the state of the balance system could be different for every different sine stimulus making it improper to combine the results. To ensure that the balance system is tested under a fixed condition, it is necessary to apply wide-bandwidth stimuli with popular possibilities being pseudorandom waveforms derived from pseudorandom ternary sequences or multisine stimuli (Davies, 1970; Peterka, 2002; Wagner et al., 2024). However, the detailed properties of the stimulus can also affect the FRF measures as demonstrated recently by showing that two pseudorandom stimuli with the same peak-to-peak amplitude but with different velocities resulted in lower sensory weight measures from the stimulus with the higher velocity (Missen et al., 2024).
3) Motor Activation parameters are known to vary as a function of body size since greater ankle torque is needed to stabilize a larger body. For comparison across individuals, it is common to normalize the Motor Activation parameters by dividing by *mgh* (mass × gravity constant × CoM height above ankle joints). For comparison across studies and within studies that are comparing more than one group, it is important to compare normalized values rather than non-normalized values. This also applies to the “Passive Mechanics” parameters if they are included in the model. Some studies report parameters that are not normalized. In this case, reported anthropometric measures can be used to normalize stiffness and damping parameters.
4) Both Sensory Weights and Motor Activation parameters have an influence on FRF gain (Campbell et al., 2022a). Therefore, an observation that a subject has a higher than normal FRF gain should not be used to conclude that this person is overweighting one sensory modality (e.g., overweighting proprioception in experimental results from a surface-tilt CSMI test) because a high FRF gain can also be caused by low Motor Activation. This is an example where the CSMI model-based interpretation of the experimental FRF is particularly useful in properly understanding the cause of a balance abnormality and avoiding the potentially erroneous conclusion that a high FRF gain, or the time-domain equivalent of a high stimulus-evoked sway, is due to an overreliance on a particular sensory modality.
5) Because the stiffness and damping parameters in the Motor Activation and Passive Mechanics components of the CSMI model make similar contributions to the net ankle torque, it can be difficult for the parameter identification procedure to reliably distinguish between corresponding active and passive parameters (Peterka, 2002; Pasma et al., 2015). If a CSMI model includes a Passive Mechanics component, a reasonable procedure is to sum the corresponding Motor Activation and Passive Mechanics stiffness and damping parameters to make a comparison to the parameters from a study that did not include a Passive Mechanics component in their model.
6) CSMI models that include Motor Activation stiffness and damping parameters (*K*_*p*_ and *K*_*d*_) + Torque Feedback and those that include Motor Activation stiffness, damping, and integration parameters (*K*_*p*_, *K*_*d*_, and *K*_*i*_) without Torque Feedback are both able to account for low frequency gain and phase results seen in experimental FRFs at frequencies down to about 0.05 Hz. However, these two different model variations have been shown to affect other model parameters (Peterka et al., 2018). Specifically, the *K*_*p*_, *K*_*d*_, and *T*_*d*_ parameters were slightly larger (3.8%, 1.7%, and 2.4%, respectively) when the Motor Activation component included *K*_*p*_, *K*_*d*_, and *K*_*i*_ parameters in the CSMI model that was used to fit the FRFs in an eyes-closed 2° surface-tilt condition while *W*_*prop*_ was only 0.8% larger.
7) CSMI models that include a Muscle Activation component (Pasma et al., 2015) will greatly reduce the estimate of the *T*_*d*_ parameter. This occurs because this component, represented by a fixed second order transfer function, accounts for some of the FRF phase lag seen at higher frequencies that would otherwise be accounted for by *T*_*d*_.
8) Only two of the 18 studies used stimuli that evoked frontal plane sway (Cenciarini et al., 2010; Peterka et al., 2011). It is not known whether CSMI parameters derived from responses to stimuli that evoked frontal plane sway would be comparable to those that evoked sagittal plane sway. However, it is likely that they would not be directly comparable since the closed-chain mechanics of the lower body (i.e., the structure formed by the two legs and the pelvis) affects the control of frontal plane sway (Scrivens et al., 2008) in comparison to the open-chain representation of body mechanics in the sagittal plane. Furthermore, the dynamics (i.e., gain and phase) of CoM sway responses to surface-tilt stimuli that evoke frontal plane sway have been shown to be greatly affected by stance width (Goodworth and Peterka, 2010) while stance width is not expected to affect sagittal plane sway.

### 4.2 Limitations, recommendations, and future directions

It is evident from studies listed in Table 1 that there is limited uniformity across studies in the CSMI test conditions applied, the amplitude of the stimuli applied, and the exact form of the CSMI model used to interpret the experimental results. Across studies that have investigated the same population groups, there is some, but not full, correspondence among the reported results. This inconsistent correspondence could be due to the different test and analysis methods applied (see Supplementary material for details) but also may be due to the small number of subjects in most studies and to differences in screening criteria used to define the patient and control group populations. Because of the preliminary nature of many of the studies reviewed the majority had a moderate to high risk of bias. There is a need for replication of studies in various patient populations to verify results from studies that included a limited number of subjects. There is also a need for understanding how conventional balance tests (e.g., mCTSIB and SOT) are related to CSMI results. All studies would benefit from knowing more about how CSMI parameters vary with age in healthy controls.

An important limitation is the availability of equipment to deliver CSMI stimulus. Most of the reviewed studies relied on custom-made equipment or on commercial equipment that allowed creation of custom stimulus profiles (e.g., SMART EquiTest CRS Balance Manager system [Natus Medical Inc., Middleton, WI, USA], discontinued). Other manufacturers have balance test equipment that can produce platform rotations (e.g., Bertec Balance Advantage [Bertec Corporation, Columbus, OH, USA]), but cooperation from the manufacturer would be required to permit custom programming of stimulus profiles with synchronous measurement of body sway and/or center of pressure displacements from which CoM displacements can be derived. Software for the CSMI analysis has been published (Peterka et al., 2018). Recently CSMI tests using visual stimuli have been developed using virtual reality systems that have been reported to give results equivalent to rotations of a physical visual surround (Assländer et al., 2023) with software required for this implementation and analysis publicly available (Assländer, 2024). This virtual reality equipment is additionally able to measure body displacements which are necessary for measurement of CoM displacements needed for FRF calculations without the need for dedicated motion analysis systems or force plates. Our group is currently working on adapting CSMI methods to be more clinically-accessible (Award no. CDMRP; HT94252510748 King PI).

Future studies could potentially apply stimuli that simultaneously evoke frontal and sagittal plane body sway using mathematically uncorrelated stimuli. A recent study (Wagner et al., 2024) utilized 2D support-surface multisine perturbations that evoked both frontal and sagittal plane sway and found that FRFs from the 2D tests were very similar to those obtain using 1D perturbations. Of course, this testing requires a platform that can generate 2D rotations.

An additional limitation is the time required to perform an individual test. Testing requires continuous presentation of repeated cycles of the pseudorandom or multisine stimulus profile. The duration of each cycle determines the lowest frequency in the FRF with many studies considering that a 20 s cycle duration provided an adequately low FRF frequency of 0.05 Hz. Peterka et al. (2018) suggested that seven cycles be used for surface tilt stimuli and 12 for visual stimuli. The first cycle is always discarded to avoid start-up transient responses so six and 11 cycles are analyzed. Therefore, each CSMI surface-tilt test takes a little more than 2 min and 4 min for visual-tilt tests, and the total test time can grow quickly when multiple test conditions are given with each test performed at more than one amplitude. But the suggested stimulus durations were based on stimuli with peak-to-peak amplitudes of 2° and 4°. Lower amplitude stimuli would likely require more cycles to obtain adequate reliability of FRFs needed for accurate parameter estimation. However, if a study is only concerned about group differences then shorter duration stimuli could be considered if subjects could not tolerate long tests. Shorter test durations would be expected to result in parameter estimates with higher variance but this could be offset by testing more subjects to maintain the ability to detect differences between groups.

Another decision in designing a CSMI protocol is whether to test at more than one stimulus amplitude and, if only testing at one amplitude, what that amplitude should be. As described above, FRF gain (and typically the associated Sensory Weight parameter) is dependent on stimulus amplitude. Testing at more than one amplitude is justified if there is an expectation that a patient group may show an abnormality in sensory reweighting compared to controls. This has been seen in patients with BVL (Peterka, 2002) and with Parkinsons disease (Feller et al., 2019). Otherwise, a peak-to-peak test amplitude of 1° appears to be a good choice for surface-tilt tests since it is likely that patients with a wide range of balance disorders would be able to perform this test without falling. Because sensitivity to visual-tilt tests is quite low, a 2° amplitude may be preferable since it would provide better signal-to-noise performance without risking a fall. Note that the mTBI studies (Campbell et al., 2025, 2022a,b) used 2° surface-tilt stimuli because their balance platform could not reliably deliver repeatable pseudorandom stimuli with 1° amplitude. However, they found that a subset of mTBI subjects with particularly low *K*_*p*_ often fell during a trial thus requiring trial repetition to obtain an adequate number of cycles for model-based analysis.

Our final recommendation is that future studies use CSMI models with the minimal number of parameters that have been shown to adequately account for experimental FRFs. A minimal model would not include the passive mechanical component since the experience from two studies (Peterka, 2002; Pasma et al., 2015) did not find it possible to obtain reliable estimates of *K*_*pas*_ and *B*_*pas*_ parameters. Additionally, we recommend not including a Muscle Activation component since this greatly affects the *T*_*d*_ estimate and limits comparison with studies that mostly have not included a Muscle Activation component. Our recommended minimal CSMI model includes only five parameters which are a sensory weight parameter (*W*_*prop*_ *or W*_*vis*_ depending on the stimulus), *T*_*d*_, and either Motor Activation including *K*_*p*_, *K*_*d*_, and *K*_*i*_ parameters or Motor Activation including *K*_*p*_ and *K*_*d*_ + Torque Feedback (*K*_*t*_).

In summary, given the above limitations in the current literature and in the implementation of CSMI testing, further studies are needed to standardize the CSMI. Future research should include replication studies with larger samples of patient groups, particularly control data across age ranges to capture potential changes related to aging. The CSMI shows promise as a tool that provides output parameters with clinical interpretability for assessing the cause of imbalance in patient populations, and trials are needed to determine if it can be implemented successfully in a clinical setting using currently available platform and/or virtual reality devices.

## 5 Conclusion

The CSMI “gray-box” model can be used to interpret experimentally evoked body sway by identifying parameters of functionally meaningful components of the balance control system. This is achieved through computerized posturography using continuous, wide bandwidth, pseudorandom perturbations of a support surface or visual surround. While variations of the CSMI-type model have been applied in different studies, generally the model is able to describe sensory weights, timing of responses to the perturbations, and Motor Activation components that account for the generation of corrective ankle torque. Testing using the CSMI-type model has been applied to a variety of patient populations, demonstrating differences in postural control from healthy controls and following interventions. However, due to lack of model and test protocol standardization, direct comparisons of results across studies remain difficult. The CSMI is a useful research and clinical tool to identify novel balance control components that could be addressed through therapies to decrease fall risk in patient populations, and to track balance control changes over time.
