# Supplementary material for: Assessment of balance control mechanisms in patient populations using perturbation-based modeling: a narrative review with clinical implications
Source: Front Hum Neurosci. 2026 May 28;20:1768417. doi: 10.3389/fnhum.2026.1768417 (PMC13253636; doi:10.3389/fnhum.2026.1768417)
Supplement: Supplementary file 1 [file Supplementary_file_1.pdf]

Supplementary Material

Assessment of Balance Control Mechanisms in Patient Populations Using Perturbation-Based Modeling: A Narrative Review with Clinical Implications

Jennifer L. Brodsky, Jae W. Lee, Laurie A. King, Robert J. Peterka

Below we list detailed information about the Central SensoriMotor Integration (CSMI) parameters, parameter estimation, and variations in the basic CSMI model shown in **Figure 1** of the main paper. This figure includes the transfer function equations of the components of the model that are represented by differential equations. The differential equations are represented in the Laplace domain where 's' is the Laplace variable. Additionally, we provide Matlab code (The Mathworks, Natick MA) that can be used to create pseudorandom stimuli that are suitable for CMSI testing.

1) The body is represented by a single segment 'Inverted Pendulum Body' that does not consider the multi-joint mechanics in a freestanding person. The input to the Inverted Pendulum Body block is ankle torque and the output variable is center-of-mass (CoM) sway angle. One study performed CSMI tests in freestanding subjects and the same subjects who were backboard constrained to sway about the ankle joints and saw no notable differences in the frequency response functions (FRFs) at frequencies up to about 2.5 Hz (Peterka, 2002). In both the freestanding and backboard conditions CoM sway angle was the output variable in the FRF analyses as it is in all CSMI experiments and models.

2) The body moment of inertia about the ankle joint (J), height of the CoM above the ankle joint (h), and body mass (m) are important parameters of the inverted pendulum body model that must be independently estimated. Body mass should not include the mass of the feet (subtracting ~2.5% of total mass to exclude the mass of the feet). The parameters J and h can be estimated using anthropometric body measurements combined with the measured body mass (Winter, 1990). The transfer function of the inverted pendulum body is $1/(Js^2 - mgh)$ where g is the gravitational constant.

3) Sensory integration is represented by a weighted summation of orientation signals from proprioception, encoding ankle joint angle, vision, encoding body tilt relative to the visual scene, and vestibular, encoding body tilt in space. The sensory weights (W_{prop}, W_{vis}, W_{vest}) represent the relative contributions of each sensory system and therefore sum to 1.0. Experiments that use surface rotations with eyes closed or open (SS/EC and SS/EO conditions) allow for the measurement of the proprioceptive system contribution, W_{prop}. Experiments that use visual scene rotations (VS/EO condition) allow for the measurement of the visual system contribution, W_{vis}. The vestibular system contribution, W_{vest}, can be inferred from tests that use surface tilt stimulation with eyes closed (SS/EC condition) where the visual contribution W_{vis} is zero. That is, $W_{vest} = 1 - W_{prop}$. W_{vest} can also be inferred in tests that use combined surface and visual tilt stimuli (SS+VS/EO condition). In

this test condition, the analysis gives an estimate of W_{prop}+W_{vis} and then W_{vest} = 1 – (W_{prop} + W_{vis}). The surface tilt with eyes open condition (SS/EO) gives a measure of W_{prop} and then the combined vestibular and visual contribution is given by W_{vest} + W_{vis} = 1 - W_{prop}. CSMI methods can be applied using surface translational stimuli where body inertia generates an ankle torque disturbance proportional to the translational acceleration. But with translation stimuli it is not possible to estimate sensory weights since all sensory systems are assumed to encode the identical body sway motion.

4) The three sensory systems are presumed to encode accurate (veridical) angular orientation cues of their respective physical variables and therefore include no dynamics and no explicit representation of the complex properties of peripheral sensory organs (e.g., stretch receptors, vestibular semicircular canals and otolith organs, retinal processes). This means that the transfer function in each of the three sensory blocks in **Figure 1B** is simply a gain multiplier of 1.0.

5) There is a single 'Time Delay' block, which represents all delays in the system including sensory transduction and transmission, central processing time, motor efferent transmission, and muscle activation delays. There is no representation of potentially different delays for the different sensory systems. The Laplace representation of a Time Delay transfer function is $e^{-T_d s}$ where T_d is the Time Delay in seconds.

6) The 'Motor Activation' block transforms sensory-derived orientation information into corrective ankle torque or to an efferent signal that is sent to the muscle if the model includes a 'Muscle Activation' block. Many CSMI-related model descriptions refer to the Motor Activation component as the 'Neural Controller'. Stabilization of an inverted pendulum minimally requires two components of corrective torque with one proportional to body sway angle and the other proportional to body sway angular velocity. The transfer function of this block is a differential equation that represents these two components with K_p being a 'stiffness' factor (units Nm/rad) that generates ankle torque proportional to the time-delayed sensed body sway angle (the input to the Motor Activation block), and K_d being a 'damping' factor (units Nms/rad) that generates ankle torque proportional to the sensed body sway angular velocity. This type of control action is commonly referred to as providing proportional + derivative (PD) control. The Laplace domain transfer function is $K_p + K_d s$.

7) An alternative Motor Activation block additionally includes an integral control factor, K_i, that generates a component of corrective ankle torque proportional to the mathematical integration of sensed body sway angle. This type of control action is designated as proportional + integral + derivative (PID) control. Inclusion of the integral component accounts for the experimentally observed FRF gain decline and phase advance that is typically seen in FRFs at stimulus frequencies below about 0.1 Hz (e.g., see **Figure 1C**). A CSMI model that includes PID control does not include the Torque Feedback mechanism shown in Figure 1A. The Laplace domain transfer function for PID control is $K_p + K_d s + K_i/s$.

8) An alternative means of accounting for the low frequency FRF gain decline and phase advance is to include a 'Torque Feedback' mechanism with PD control in the Motor Activation block. The Torque Feedback block includes a differential equation that represents a mathematical integration of the sensed ankle torque with a gain factor K_t. The transfer function for the Torque Feedback block is K_t/s. In some experiments that investigated very low frequency behavior, the Torque Feedback transfer function is represented by a lowpass filter with a long time constant (Cenciarini & Peterka, 2006; Missen et al., 2025). However, in typical experiments that identify FRFs at the

lowest frequency of 0.05 Hz, a CSMI model with PID or PD + Torque Feedback has a similar ability to account for low frequency behavior (Peterka et al., 2018).

9) The Torque Feedback contributes to overall ankle torque via a positive feedback mechanism in contrast to the negative feedback mechanism that processes information from proprioception, visual, and vestibular sensory systems. This Torque Feedback mechanism will slowly move the body to an upright orientation following a balance perturbation that causes a tilt in body orientation away from upright as can be caused by a tilt of the stance surface or the visual scene. In contrast, PID control will move the body into alignment with the tilted surface or visual scene but not to upright. Therefore, these two mechanisms do not predict the same low frequency behavior of the balance system, but this will only be evident in experiments that investigate very low frequency behavior (Missen et al., 2025).

10) A CSMI-type model may include a component representing Muscle Activation dynamics by a fixed second-order differential equation (Pasma et al., 2015). Its transfer function has been represented by the equation $\omega^2/(s^2 + 2\beta\omega s + \omega^2)$ with fixed parameters $\omega = 5\pi$ rad/s (the 'natural frequency' parameter) and $\beta = 0.7$ (the damping coefficient). The input to the Muscle Activation block is the output of the Motor Activation block and the output is an active ankle torque that is summed with a passive torque contribution if the model includes a 'Passive Mechanics' component. The summed ankle torque is the input to the inverted pendulum body. Inclusion of a Muscle Activation component accounts for some of the phase lag observed in the overall stimulus/response FRF. This results in smaller values of the estimated Time Delay parameter T_d compared to a CSMI model that does not include Muscle Activation dynamics.

11) The CSMI model does not include an explicit representation of muscle/tendon mechanics, but some CSMI models do include a simple representation of the passive/intrinsic contributions to ankle torque due to muscle/tendon stretch from ankle angle changes. In **Figure 1B**, this contribution is represented in the Passive Mechanics block. This block represents a differential equation similar to the Motor Activation PD control block and includes a stiffness factor, K_{pas}, and damping factor, B_{pas}, that generate ankle torque in proportion to ankle joint angle and angular velocity, respectively, without any Time Delay. The Laplace domain transfer function is $K_{pas} + B_{pas}s$.

12) The first study to develop a CSMI-type model explored whether Passive Mechanics parameters could be reliably estimated and found mixed results (Peterka, 2002). When reliable estimates seemed possible, about 15% of the total ankle torque was attributable to the passive mechanism. However, when estimates seemed less reliable, the distinction between K_p and K_{pas} and between K_d and B_{pas} parameters was not clear since each pair of parameters contributed similarly to ankle torque production. As discussed in a study that performed a sensitivity analysis on parameters of a CSMI model (Pasma et al., 2017), variations in some parameters have similar effects on the model transfer function making it difficult to uniquely identify the contributions from different mechanisms with an example being K_p and K_{pas}. This lack of uniqueness justifies the recommendation to not include some components of the model, such as the Passive Mechanics block, in order to obtain more reliable estimates of the remaining parameters of a simplified CSMI model. The caveat is that the simplified model still needs to account well for the experimental FRF.

13) There is no representation of a stretch reflex contribution to ankle torque in the CSMI model. A potential simple stretch reflex contribution could be represented by a transfer function of the form $(K_{sr} + B_{sr}s)e^{-T_{sr}s}$ with the input being the ankle joint angle (CoM angle minus Surface tilt angle) and the output is the ankle torque contribution due to the stretch reflex that sums with the passive

torque generated by the Passive Mechanics and the active torque generated by Motor Activation. K_{sr}, B_{sr}, T_{sr} are stretch reflex stiffness, damping, and Time Delay parameters, respectively. However, similar to the discussion concerning the Passive Mechanics mechanism, there is a high likelihood that stretch reflex parameters would be difficult to uniquely distinguish from parameters of the Motor Activation mechanism.

14) All differential equations in the model are expressed in the Laplace domain, which allows for algebraic calculations of the transfer function relating the stimulus to CoM response. The parameters of this transfer function can be estimated using mathematical optimization methods to adjust them to optimally match the experimental FRF. The Matlab function 'fmincon' available in the Optimization Toolbox (Mathworks, Natick MA, USA) can be used for this purpose. The choice of the error function to be minimized by the optimization procedure is defined as some function that quantifies the difference between the FRF from the model fit and the experimental FRF. Different error functions have been used in studies, and this will have some influence on the values of the estimated parameters.

15) When all components of the CSMI model are combined into a single equation representing a transfer function that relates the CoM sway angle (the evoked response) to a surface-tilt stimulus (SS/EC or SS/EO conditions), a visual tilt stimulus (VS/EO condition), or combined surface plus visual stimuli (SS+VS/EO), respectively, the equations are:

$$\frac{CoM}{Surface\ Stimulus} = \frac{PM \cdot B + W_{prop} \cdot MA \cdot TD \cdot B}{1 - MA \cdot TD \cdot TF + PM \cdot B + MA \cdot TD \cdot B}$$

$$\frac{CoM}{Visual\ Stimulus} = \frac{W_{vis} \cdot MA \cdot TD \cdot B}{1 - MA \cdot TD \cdot TF + PM \cdot B + MA \cdot TD \cdot B}$$

$$\frac{CoM}{Surface + Visual\ Stimulus} = \frac{PM \cdot B + \left(W_{prop} + W_{vis}\right) \cdot MA \cdot TD \cdot B}{1 - MA \cdot TD \cdot TF + PM \cdot B + MA \cdot TD \cdot B}$$

Where the symbols *PM*, *MA*, *TF*, *B*, and *TD* are the transfer functions representing Passive Mechanics, Motor Activation, Torque Feedback, Inverted Pendulum Body, and Time Delay, respectively. If the Muscle Activation component is included in the model, then the Motor Activation transfer function should be multiplied by the Muscle Activation transfer function. If it is desired that the model not include a passive contribution to corrective torque, then set *PM* to zero in the above equations. If PID control is chosen to represent the Motor Activation component, then *TF* should be set to zero. After the individual component Laplace domain transfer functions are substituted into the above CSMI model equations, the substitution for the Laplace variable $s = j2\pi f$ (where j is the imaginary number $\sqrt{-1}$ and f is the stimulus frequency in Hz) allows for the calculation of the model's FRF that can be expressed as gain and phase values as a function of frequency as shown in **Figure 1C**.

16) Below is example Matlab code that generates a pseudorandom stimulus suitable for CSMI testing. The stimulus is based on a mathematically defined 'm-sequence' of numbers that have desirable properties when used to create stimuli for system identification (Davies, 1970). A wide variety of pseudorandom sequences can be generated using a Matlab function named 'mseq' that is freely available (Buracas, 2003). All the studies summarized in the main paper used stimuli based on a ternary m-sequence (i.e., pseudorandom ternary sequence, PRTS) with the pseudorandom sequence having values -1, 0, or +1 generated by the 'mseq' function or other equivalent functions used by various investigators. The PRTS was processed by the Matlab code included below to generate stimuli. Examples of one cycle of a pseudorandom stimulus are shown in **Figure 1B**.

Example Matlab code:

```
Amp_pp = 2;  % Desired peak-to-peak amplitude of integrated PRTS waveform
ppdt = 25;       % Each PRTS sequence value is repeated ppdt times
cycles = 7;      % Desired number of PRTS cycles
%
% Generate the PRTS maximal length sequence using 'mseq' function
%   -  Sequence length is 3^4-1 = 80
%   -  The sequence is a vector of numbers with values +1, 0, or -1
%   -  Shift value determines the start of the PRTS sequence. A shift value of
%        59 results in an asymmetric final stimulus waveform that moves more in
%        one direction than the opposite direction.
%   -  Shift values of 1, 22, 79 give approximately symmetric stimuli

ms = mseq(3,4,59,1);

% Integrate the PRTS sequence with ppdt points per sequence value
i = length(ms);                  % Determine the length of the m-sequence
msi = zeros(1, ppdt * i);  % Initialize the integrated PRTS sequence
for j = 1:i
  for jj = 1:ppdt
    msi(jj+(j-1)*ppdt+1) = msi(jj+(j-1)*ppdt)+ms(j);
  end
end
msi = msi(1:ppdt*i);                   % delete the last point which repeats the first point

msi = msi/(max(msi)-min(msi));    % normalize to 1 peak-to-peak
ms2 = Amp_pp*msi;                    % scale to the desired peak-to-peak amplitude
PRTSstim = ms2;
for i = 1:(cycles-1)                  % Repeat to generate desired number of cycles
    PRTSstim = [PRTSstim ms2];
End

samprate=100;                          % Sample rate of stimulus delivery system
t = (0:length(PRTSstim)-1) / samprate; % Generate a time vector for plotting
plot(t, PRTSstim)
title('PRTS stimulus: 7 cycles, 20 s cycle duration, 2 deg peak-to-peak amplitude')
ylabel('Stimulus tilt angle (deg)'); xlabel('Time (s)')
```

References

Cenciarini, M. & Peterka, R.J.: Stimulus-dependent changes in the vestibular contribution to human postural control. Journal of Neurophysiology, 95(5):2733-2750, 2006.

Davies, W. D. T. (1970) *System Identification for Self-Adaptive Control*. London: Wiley-Interscience.

Buracas, G.: https://www.mathworks.com/matlabcentral/fileexchange/3083-m-sequence-generation-program

Missen, K.J., Carpenter, M.G, & Lorenz Assländer, L.: Slow dynamics of human balance control. Scientific Reports, 15:27593, 2025.

Pasma, J.H., Engelhart, D., Maier, A.B., Schouten, A.C., van der Kooij, H., Meskersm C.G.: Changes in sensory reweighting of proprioceptive information during standing balance with age and disease. Journal of Neurophysiology, 114(6):3220–33, 2015.

Pasma, J.H., Boosntra, T.A., van Kordelaar, J., Spyropoulou, V.V., Schouten, A.C.: A sensitivity analysis of an inverted pendulum balance control model. Frontiers in Computational Neuroscience, 11:99, 2017.

Peterka, R.J.: Sensorimotor integration in human postural control. Journal of Neurophysiology, 88(3):1097-1118, 2002.

Peterka, R.J., Murchison, C.F., Parrington, L., Fino, P.C., King, L.A.: Implementation of a central sensorimotor integration test for characterization of human balance control during stance. Frontiers Neurology 9:1045, 2018.

Winter, D.A. (1990) *Biomechanics and Motor Control of Human Movement*. New York: Wiley.
